# Genetic variants and polygenic risk scores associated with paroxysmal atrial fibrillation in the Japanese population

**DOI:** 10.1371/journal.pone.0344360

**Published:** 2026-05-04

**Authors:** Megumi Shiomi, Yuki Nagata, Takeaki Sudo, Kentaro Takahashi, Chihiro Higuchi, Kensuke Ihara, Ken Asada, Yasuaki Tanaka, Yasuteru Yamauchi, Takeshi Sasaki, Hitoshi Hachiya, Yasushi Imai, Hideo Fujita, Tetsuo Sasano, Tetsushi Furukawa, Toshihiro Tanaka

**Affiliations:** 1 Department of Human Genetics and Disease Diversity, Graduate School of Medical and Dental Sciences, Institute of Science Tokyo, Tokyo, Japan; 2 Bioresource Research Support Center, Institute of Science Tokyo, Tokyo, Japan; 3 Department of Educational Media Development, Institute of Science Tokyo, Tokyo, Japan; 4 Artificial Intelligence Center for Health and Biomedical Research, National Institutes of Biomedical Innovation, Health and Nutrition, Osaka, Japan; 5 Department of Bio-informational Pharmacology, Medical Research Institute, Institute of Science Tokyo, Tokyo, Japan; 6 Department of Cardiovascular Medicine, Institute of Science Tokyo, Tokyo, Japan; 7 AI Medical Engineering Team, RIKEN Center for Advanced Intelligence Project, Tokyo, Japan; 8 Division of Medical AI Research and Development, National Cancer Center Research Institute, Tokyo, Japan; 9 Cardiovascular Center, Yokosuka Kyosai Hospital, Kanagawa, Japan; 10 Department of Cardiology, Yokohama City Minato Red Cross Hospital, Kanagawa, Japan; 11 Department of Cardiology, Heart Rhythm Center, National Hospital Organization Disaster Medical Center, Tokyo, Japan; 12 Cardiovascular Center, Tsuchiura Kyodo General Hospital, Ibaraki, Japan; 13 Division of Clinical Pharmacology, Department of Pharmacology, Jichi Medical University, Tochigi, Japan; 14 Division of Cardiovascular Medicine, Saitama Medical Center, Jichi Medical University, Saitama, Japan; Calico: Calico Life Sciences LLC, UNITED STATES OF AMERICA

## Abstract

Early-stage diagnosis of paroxysmal atrial fibrillation (PAF) is challenging owing to its asymptomatic nature. However, the genetic factors underlying PAF and predictive utility of polygenic risk scores (PRSs) for PAF in Asian populations remain elusive. We aimed to explore the PAF-associated genetic variants in a Japanese cohort and evaluate the predictive performance of PAF-specific PRSs. This study included 2,604 participants. Following exclusion, quality control, and genotype imputation, a genome-wide association study (GWAS) was conducted. The predictive performance of 30 sets of PRS models constructed across various thresholds was evaluated using three machine learning methods. Model performance was assessed using area under the curve (AUC) and SHapley Additive exPlanations (SHAP). The GWAS using 1,038 PAF cases and 744 controls identified 82 genome-wide significant variants (*P* < 5 × 10^−8^), all on chromosome 4q25. Of these, 80 variants clustered upstream of *PITX2*, and two were located in *LINC01438*. Fine mapping identified two independent intergenic signals, with rs2200732 as the lead single-nucleotide polymorphism. The best PRS-only model achieved an AUC of >0.70, which was improved up to 0.737 in additive models incorporating both PRS and clinical variables. SHAP analysis consistently ranked PRS as the most influential predictor among the clinical variables included in this study. These results suggest that genetic risk, particularly at the established 4q25/*PITX2* locus, contributes substantially to PAF susceptibility in this Japanese cohort and that PRS may improve early risk stratification when integrated with clinical risk factors.

## Introduction

Paroxysmal atrial fibrillation (PAF), the most common clinical subtype of atrial fibrillation (AF), accounts for approximately 50% of all AF cases in Japan [1]. Clinically, AF is classified as paroxysmal, persistent, long-standing persistent, or permanent. PAF is characterized by its intermittent and self-terminating nature, resolving spontaneously within 7 days of onset. Although PAF often initially presents with brief episodes, it can progress to persistent or permanent AF over time [2], leading to a worsening prognosis as the disease advances [3]. The majority of patients hospitalized for stroke and diagnosed with new-onset AF reportedly have asymptomatic PAF [4]. Moreover, PAF is reported to be more prevalent than persistent AF among patients with a history of stroke or transient ischemic attack [5]. These findings highlight the need for early detection and intervention. However, diagnosing PAF remains challenging owing to its intermittent, short-lived, and frequently asymptomatic presentation.

The development of AF, including PAF, is associated with advanced age and established risk factors, such as smoking, alcohol consumption, obesity, hypertension, diabetes, and cardiovascular disease [6]. In addition to these clinical and environmental factors, genetic predisposition plays a significant role in AF pathogenesis [7–11]. AF can occur in the absence of conventional risk factors, suggesting familial or heritable forms [7].

Since the first genome-wide association study (GWAS) on AF, over 100 susceptibility loci have been identified across multiple ethnic populations [8–11]. Among these, the 4q25 locus near *PITX2* is one of the most consistently replicated genetic signals for AF susceptibility [8–11]. *PITX2* encodes a transcription factor implicated in atrial development and electrophysiological regulation [10]. Functional studies, including animal models and human iPSC-derived cardiomyocytes, further suggest that reduced *PITX2* activity may alter the electrophysiological properties of cardiomyocytes [12,13]. The genetic architecture of AF has been reported to differ between Japanese and European populations [11]. In Japanese cohorts, associations at the 4q25/*PITX2* locus have also been confirmed, and six additional loci (*KCND3*, *PPFIA4*, *SLC1A4–CEP68*, *HAND2*, *NEBL*, and *SH3PXD2A*) have been identified [11]. Several of these genetic loci are specific to Japanese and East Asian populations and have not been consistently observed in studies of populations with European ancestry, highlighting genetic diversity between populations. These ancestry-dependent differences underscore the need for gene discovery and risk prediction using population-matched cohorts. However, most previous studies have treated AF as a single phenotype. As a result, the specific contributions of established AF-associated loci, such as *PITX2*, to PAF remain poorly understood.

Polygenic risk scores (PRSs), which aggregate the effects of multiple disease-associated single-nucleotide variants, have emerged as valuable tools for estimating the genetic risk of an individual. Khera et al. developed PRSs for five common diseases, including AF, and demonstrated that these scores can identify individuals with risk comparable to that conferred by monogenic variants [14]. Although PRSs for AF show limited predictive performance when used in isolation, their performance improves when combined with clinical risk models [15]. Most AF PRSs have been developed in populations of European ancestry [15], although several recent studies have constructed and validated AF PRSs in Japanese cohorts [16–18]. However, these studies have primarily focused on general AF phenotypes without distinguishing PAF from persistent AF. Additionally, the utility of PRSs specifically for PAF remains largely underexplored, despite its clinical significance and high prevalence. As PAF may represent a distinct phenotype within AF, evaluating its genetic features and predictive models separately is essential to advance precision medicine approaches.

In this study, we aimed to uniquely target PAF, which may have distinct genetic underpinnings and clinical implications. We conducted a GWAS in a Japanese population to identify susceptibility loci associated with PAF. Furthermore, we constructed PRSs by aggregating the effects of identified risk variants using a clumping and thresholding approach. These PRSs were evaluated using multiple machine learning algorithms to assess their predictive utility alone and in combination with clinical variables and gene–environment interaction terms. To enhance interpretability and facilitate clinical translation, we used SHapley Additive exPlanations (SHAP) to quantify the relative contributions of genetic and clinical predictors [19]. Overall, this study aimed to evaluate whether PRSs can predict the risk of PAF in a Japanese cohort and to identify PAF-relevant susceptibility loci. Our findings indicate that these aims were achieved.

## Materials and methods

### Study participants

In total, 2,604 participants were enrolled between March 1, 2020 and December 31, 2021, including inpatients and outpatients from the Department of Cardiology of Tokyo Medical and Dental University Hospital, Jichi Medical University Hospital, Yokohama City Minato Red Cross Hospital, Jichi Medical University Saitama Medical Center, Yokosuka Kyosai Hospital, National Hospital Organization Disaster Medical Center, and Tsuchiura Kyodo General Hospital. This study was conducted in Japanese subjects, and participants were classified as PAF cases or controls; those in the control group were unrelated individuals without any history of AF, recruited from the same institutions. AF was defined as an electrocardiogram (ECG) recording lasting over 30 s, while PAF was defined as AF that spontaneously terminated within 7 days of onset, in accordance with the Japanese Circulation Society/Japanese Heart Rhythm Society guidelines [20]. All participants underwent ECG assessments at their respective institutions. PAF cases were identified based on physician diagnoses with ECG documentation. Controls were defined as individuals without a documented history of AF/PAF and without AF documented on the ECG performed at the participating institutions. Clinical exclusion criteria were: (1) missing clinical information, (2) AF other than PAF, (3) history of heart failure (HF), (4) coronary artery disease (CAD), or (5) valvular heart disease. HF, CAD, and valvular heart disease, an organic cardiac condition that directly affects AF [6], were excluded to avoid potential confounding in comparisons between PAF cases and controls from the same cardiology departments. Collected clinical information included age, body mass index (BMI), cardiometabolic parameters at sample collection, age at PAF diagnosis (for PAF cases), sex, smoking and alcohol consumption (current drinker vs. non-drinker), histories of hypertension (HT), diabetes mellitus (DM), dyslipidemia (DL), cerebral infarction (CI), and family history of AF. In addition to medical records, HT was defined as a systolic blood pressure ≥140 mmHg or diastolic blood pressure ≥90 mmHg or the use of antihypertensive medications [21]. DM was defined as a fasting plasma glucose level ≥126 mg/dL, random plasma glucose level ≥200 mg/dL, or use of antidiabetic medications [22]. DL was defined as low-density lipoprotein cholesterol ≥140 mg/dL, high-density lipoprotein cholesterol <40 mg/dL, triglycerides ≥150 mg/dL in the fasting state or ≥175 mg/dL in the non-fasting state, or use of lipid-lowering medications [23].

This study was approved by the institutional review boards of all participating institutions. Ethical approval for the overall study was granted by the Ethics Committee of Tokyo Medical and Dental University (No. O2019-006), and the study was conducted in accordance with the principles of the Declaration of Helsinki. Written informed consent was obtained from all participants.

### Single-nucleotide polymorphism (SNP) genotyping, quality control, and genetic imputation

Genomic DNA was extracted from peripheral blood samples and genotyped using the Infinium Asian Screening Array-24 v1.0 BeadChip (Illumina, Inc., San Diego, CA, USA), which covers 659,184 common SNPs, at Macrogen Japan (Tokyo, Japan). Genotype calling was performed using GenomeStudio v2.0 (Illumina, Inc.). Quality control of the raw data was conducted using PLINK v1.9 [24] at the sample and SNP levels. Samples were excluded for sex discrepancy, call rate <97%, excess heterozygosity (heterozygosity rate ± 3 standard deviations from the mean), or relatedness (PI_HAT > 0.185). Population stratification was assessed using principal component analysis, comparing the first four principal components with five reference populations (Africans, Americans, South Asians, East Asians, and Europeans) from the 1000 Genomes Project [25]. SNPs were excluded if they had a call rate <95%, Hardy–Weinberg equilibrium *P*-value <1.0 × 10^−6^, or minor allele frequency (MAF) <0.01. Association analyses were performed using autosomal SNPs. Pre-phasing was performed using SHAPEIT2 [26] and imputation using Minimac3 [27], with the 1000 Genomes Phase 3 reference panel [25]. Post-imputation, SNPs were filtered for imputation quality (Rsq < 0.3), MAF < 0.01, and duplications. Accordingly, our association analyses focused on variants with a MAF of ≥0.01, and rare-variant association tests were not performed.

### GWAS analysis

GWAS was conducted using logistic regression in PLINK [24], adjusting for covariates including sex, smoking status, alcohol consumption, history of HT, DM, DL, and CI, and family history of AF (maternal, paternal, and other relatives). We did not include BMI or cardiometabolic laboratory measurements at the time of sample collection as covariates because these measurements may be influenced by ongoing treatments and, for some cases, may have been obtained after the PAF diagnosis. Instead, we adjusted for major comorbidity histories defined by standardized clinical criteria. Genome-wide significance was defined as *P* < 5 × 10 ⁻ ⁸ and suggestive significance as *P* < 1 × 10 ⁻ ⁵. SNP annotations were obtained from SNPnexus [28] and dbSNP [29]. To identify lead SNPs, the Functional Mapping and Annotation (FUMA) platform v1.5.2 [30] was used, incorporating GWAS summary statistics and linkage disequilibrium (LD) data from the 1000 Genomes Phase 3 East Asian panel [25]. This analysis mapped GWAS-identified SNPs to 18,900 protein-coding genes and calculated *P*-values for each gene using multi-marker analysis of genomic annotation (MAGMA) v1.6 [31], implemented using FUMA [30]. A Bonferroni correction was applied to determine statistical significance, with the significance threshold set at a *P*-value < 2.6 × 10^−6^ (0.05/18,900). Independent significant SNPs were identified as those with a *P* < 5 × 10 ⁻ ⁸ and pairwise LD *r*^2^ < 0.6. Lead SNPs were selected as those among these with pairwise LD *r*^2^ < 0.1. Annotations were based on the GRCh37 (hg19) genome build. Functional annotation of lead SNPs included evaluation of the combined annotation-dependent depletion (CADD) score (deleteriousness) [32], the RegulomeDB score (regulatory potential) [33], expression quantitative trait loci (eQTL), and three-dimensional chromatin interactions (Hi-C data) [34]. The false discovery rate (FDR) was applied to adjust for multiple comparisons. Lead SNPs identified using FUMA [30] were subsequently used as covariates in conditional analysis performed in PLINK [24] to assess the independence of genome-wide significant insertions and deletions (INDELs) located within the same loci. Regional plots were generated using LocusZoom to visualize the LD structures surrounding significant variants [35].

### PRS construction

**Fig 1:**
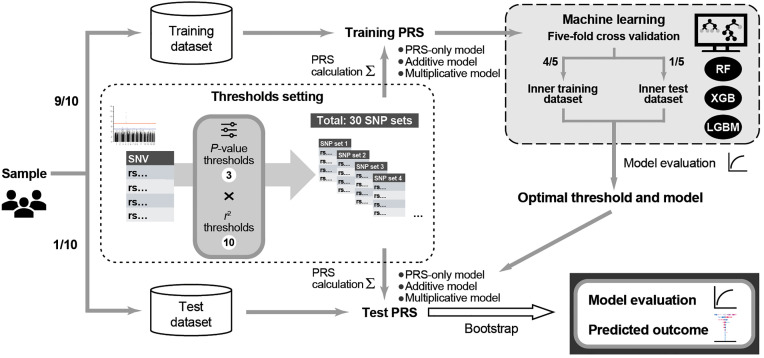
Overview of the Study Design for PRS Construction and Evaluation using Machine Learning. The entire dataset was randomly split using stratified sampling into training (90%) and test (10%) datasets to maintain the case–control ratio. Within the training dataset, 30 sets of PRS models were constructed using all combinations of three *P*-value threshold settings (5 × 10 ⁻ ⁸, 1 × 10 ⁻ ⁵, and 1 × 10 ⁻ ⁴) and 10 linkage disequilibrium (*r*²) clumping threshold settings (no clumping and 0.9 to 0.1) based on summary statistics from a published AF GWAS. For each PRS set, three machine learning models—RF, XGB, and LGBM—were trained using five-fold stratified cross-validation. The best PRS threshold setting and model combination was selected based on the mean cross-validated area under the curve and evaluated on the test dataset using 1,000 bootstrap resamples. Three types of models were developed: PRS-only, additive (PRS + clinical variables), and multiplicative (additive + interaction terms). To interpret model predictions, SHapley Additive exPlanations values were computed for the additive and multiplicative models to estimate the contribution of each feature to prediction outcomes. AF, atrial fibrillation; LGBM, light gradient boosting machine; PRS, polygenic risk score; RF, random forest; XGB, extreme gradient boosting.


Before PRS calculation, the entire dataset was randomly split into a training dataset (90%) and a test dataset (10%) while preserving the case–control ratio through stratified sampling. Given the modest sample size, we adopted a 90/10 split to maximize the samples available for model development while maintaining an independent dataset for final evaluation. This split was performed once and fixed throughout all subsequent analyses to avoid information leakage. PRS for PAF were constructed using SNP effect size estimates (summary statistics) from a published AF GWAS [11]. The rationale for selecting this reference GWAS is detailed in the Results section. The framework of this study is shown in Fig 1. To evaluate the effect of GWAS thresholds and LD clumping on PRS, we created 30 SNP sets representing all combinations of three *P*-value thresholds (5 × 10 ⁻ ⁸, 1 × 10 ⁻ ⁵, and 1 × 10 ⁻ ⁴) and 10 squared correlation (*r*^2^) thresholds for clumping (no clumping and 0.9 to 0.1) within a 250-kb window. For each SNP set in the training and test datasets, we calculated:

$$PRS_{GW\;\;AS}=\;\sum_{i=\text{1}}^{n}\overset{GWAS}{\underset{i}{\beta }}Gi$$

where $\overset{GWAS}{\underset{i}{\beta }}$ is the log odds ratio (effect size) for SNP_*i*_, which represents the summary statistics published in AF GWAS [11], *G*_*i*_ is the genotype count (0, 1, or 2), and *n* is the number of SNPs included, representing the data evaluated in this study.

### Machine learning-based evaluation of PRS

In the training dataset, three ensemble-based machine learning models—random forest (RF), extreme gradient boosting (XGB), and light gradient boosting machine (LGBM)—were evaluated owing to their ability to model non-linear relationships, robustness to feature correlation, and compatibility with SHAP-based interpretation. These three algorithms were selected a priori as representative tree-ensemble methods for tabular clinical/genetic data, facilitating a consistent SHAP interpretability workflow using TreeExplainer across models. Single decision trees and non-tree-based models were not evaluated in this analysis. Three model types were evaluated: 1) PRS-only model; 2) additive model: PRS + clinical variables (sex, smoking, alcohol consumption, history of HT, DM, DL, and CI, and family history of AF); and 3) multiplicative model: additive model + interaction terms (PRS × each clinical variable). Interaction terms were specified a priori to assess potential effect modification of genetic risk by major clinical factors known to be relevant to AF. To limit model complexity and reduce the risk of overfitting, interactions were restricted to covariates with complete data (no missingness). Hyperparameter tuning was performed using Bayesian optimization with Optuna (Tree-structured Parzen Estimator; seed = 42) under five-fold stratified cross-validation, maximizing cross-validated area under the curve (CV-AUC); we ran 100 trials per algorithm per feature set. Hyperparameter search spaces for RF, XGB, and LGBM are provided in S1 Table. The model with the highest mean CV-AUC across the five folds was selected and tested on the test dataset. To assess robustness, 95% confidence intervals (95% CI) were calculated using 1,000 non-parametric bootstrap resamples from the test dataset. Performance metrics included AUC, area under the precision–recall curve (AUPRC), and F1 score, defined as the harmonic mean of precision and recall. As a sensitivity analysis, we also evaluated clinical-covariate–only models (excluding PRS) using the same fixed training/test split and evaluation pipeline.

### SHAP-based feature interpretation

To enhance model transparency and assess feature contribution, SHAP values were calculated to estimate the marginal contribution of each feature to individual predictions [19]. The TreeExplainer method was used for each model (RF, LGBM, and XGB). Summary plots were generated to visualize the distribution of feature importance across all samples. This interpretability analysis was applied to additive and multiplicative models.

### Statistical analysis

Continuous variables are summarized as mean ± standard deviation, and categorical variables as counts and percentages. Differences between PAF cases and controls were tested using Student’s *t*-test for continuous variables and Fisher’s exact test for categorical variables. General statistical analyses were conducted using R v4.4.1. All statistical tests were two-sided at the 0.05 significance level, unless stated otherwise. Machine learning and SHAP analyses were performed in Python v3.12.9 using scikit-learn v1.6.1 (including RF), LGBM v4.6.0, XGBoost v3.0.0, and Optuna v4.3.0.

## Results

### Quality control, imputation, and clinical characteristics

Of the 2,604 participants enrolled, 764 were excluded based on clinical criteria, and an additional 58 were removed during genotype-based quality control. Additional details on the specific exclusion reasons and quality control thresholds are provided in S1 Fig. Finally, 1,782 participants (1,038 PAF cases and 744 controls) were included in the GWAS and PRS analyses. At the SNP level, 215,273 variants were removed based on quality control filters, leaving 443,911 variants for the downstream analysis. After imputation filtering, 8,094,202 variants were selected for GWAS (S1 Fig). Baseline characteristics of the participants are summarized in Table 1. PAF cases were more likely to be male (*P* = 0.009), older at the time of sample collection (66.4 ± 11.5 vs. 64.9 ± 13.6 years; *P* = 0.02), and alcohol consumers (*P* < 0.001) and have a family history of AF (maternal: *P* < 0.001; paternal: *P* = 0.002; other relatives: *P* < 0.001) than controls. No significant differences were observed for smoking or histories of HT, DM, DL, or CI. BMI at sample collection was comparable between PAF cases and controls. Age at sample collection was available for all participants; in contrast, age at PAF diagnosis was recorded only for a subset of cases. Among all PAF cases (n = 1,038), age at PAF diagnosis was available for 734 patients (70.7%). In this subset, the mean age at diagnosis was 65 ± 12 years, and 65% were aged ≥60 years.

**Table 1:** Clinical Characteristics of the Cases and Controls.

Variable	Cases	Controls	OR (95% CI)	*P*-value
N	1,038	744		
Female, %	34	40.1	0.77 (0.63–0.94)	0.009
Age at sample collection, years (mean ± SD)	66.4 ± 11.5	64.9 ± 13.6		0.02
BMI at sample collection, kg/m^2^ (mean ± SD)	24.0 ± 3.6	24.1 ± 3.8		0.35
SBP at sample collection, mmHg (mean ± SD)	131.6 ± 17.9	130.3 ± 17.6		0.13
DBP at sample collection, mmHg (mean ± SD)	78.6 ± 12.7	76.4 ± 12.6		0.0003
LDL at sample collection, mg/dL (mean ± SD)	108.0 ± 39.1	98.6 ± 46.6		0.000004
HDL at sample collection, mg/dL (mean ± SD)	57.0 ± 22.9	55.3 ± 26.1		0.15
TG at sample collection, mg/dL (mean ± SD)	135.1 ± 99.6	124.3 ± 93.9		0.02
FBS at sample collection, mg/dL (mean ± SD)	100.9 ± 49.8	94.7 ± 54.1		0.01
Smoking, %	49.8	48	1.08 (0.89–1.30)	0.47
Alcohol consumption, %	54.5	44.2	1.51 (1.25–1.84)	<0.001
Hypertension, %	65.5	67.5	0.92 (0.75–1.12)	0.42
Diabetes mellitus, %	25.9	30	0.82 (0.66–1.01)	0.06
Dyslipidemia, %	56.2	57.7	0.94 (0.77–1.14)	0.56
Cerebral infarction, %	3.8	2.8	1.34 (0.76–2.43)	0.29
Maternal family history of AF, %	2.6	0.5	4.94 (1.71–19.49)	<0.001
Paternal family history of AF, %	1.9	0.3	7.28 (1.76–65.53)	0.002
Other family history of AF, %	2.5	0.3	9.53 (2.37–83.21)	<0.001

AF, atrial fibrillation; BMI, body mass index; CI, confidence interval; DBP, diastolic blood pressure; FBS, fasting blood sugar; HDL, high-density lipoprotein cholesterol; LDL, low-density lipoprotein cholesterol; OR, odds ratio; SBP, systolic blood pressure; SD, standard deviation; TG, triglycerides. *P*-values were calculated using Student’s *t*-test or Fisher’s exact test implemented in R v4.4.1.

### GWAS analysis

**Fig 2:**
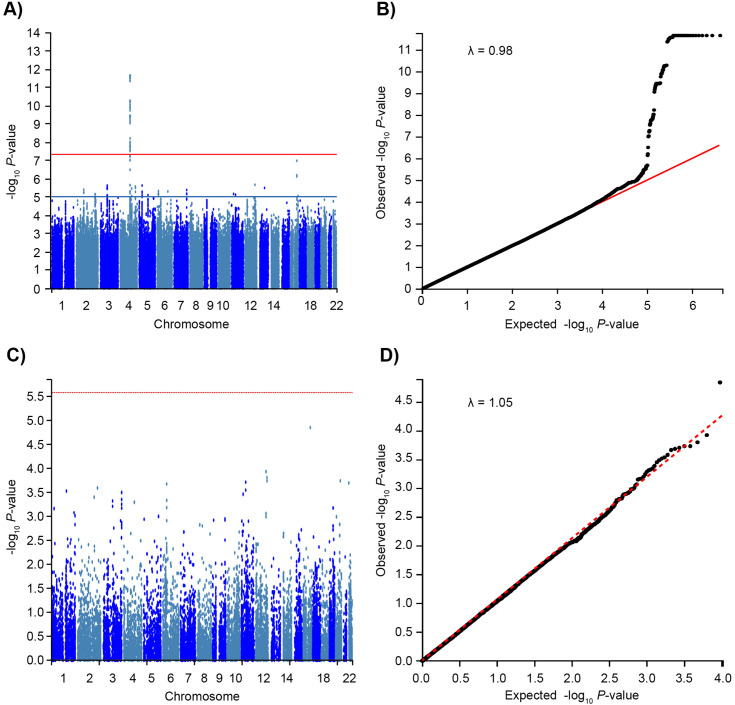
GWAS Analysis for PAF. **(A)** Manhattan plot of the SNP-based GWAS. The X‐axis represents the chromosomal location, and the Y‐axis represents the − log_10_ *P*‐value. The red line indicates the genome-wide significance threshold of *P* = 5 × 10^−8^ and the blue line indicates the suggestive threshold of *P* = 1 × 10^−5^. **(B)** Quantile–quantile plot of SNP-based GWAS. The red diagonal line represents the expected distribution of *P*-values under the null hypothesis of no association. **(C)** Manhattan plot of the gene-based GWAS. The X‐axis represents the chromosomal location, and the Y‐axis represents the − log_10_ *P*‐value. The red dashed line indicates the statistical significance threshold of *P* = 2.6 × 10^−6^, which is Bonferroni-corrected for the 18,900 protein-coding genes. **(D)** Quantile–quantile plot of gene-based GWAS. The red dashed diagonal line represents the expected distribution of *P*-values under the null hypothesis of no association. GWAS, genome-wide association study; PAF, paroxysmal atrial fibrillation; SNP, single-nucleotide polymorphism.

GWAS identified 82 genome-wide significant variants (*P* < 5 × 10 ⁻ ⁸), all located on chromosome 4q25 (Fig 2A, S2 Table). An additional 78 variants showed suggestive significance (*P* < 1.0 × 10 ⁻ ⁵; S3 Table). A quantile-quantile plot indicated minimal genomic inflation (λ = 0.98), suggesting limited population stratification (Fig 2B). Gene-based MAGMA [31] analysis revealed no genes reaching genome-wide significance (threshold *P* < 2.6 × 10 ⁻ ⁶; Fig 2C), with λ = 1.05 (Fig 2D). Of the 82 variants, 72 were SNPs and 10 were INDELs. Seventy SNPs and all ten INDELs were intergenic, located upstream of *PITX2*, whereas two SNPs were within *LINC01438*, a long intergenic non-coding RNA. Using FUMA [30], two independent significant intergenic SNPs (rs2200732 and rs13122916) were identified, with rs2200732 designated as the lead SNP (S2 Fig). Functional annotation was performed for the two independent significant SNPs, rs2200732 and rs13122916. However, neither SNP showed deleterious potential based on CADD [32] or RegulomeDB scores [33], nor were they associated with *PITX2* expression in cis-eQTL analyses. Chromatin interaction analysis identified three additional genome-wide significant SNPs—rs7434417, rs1906591, and rs6843082—in predicted enhancer regions that physically interacted with *PITX2* regulatory elements (FDR < 1.0 × 10 ⁻ ^6^). These interactions were observed in mesenchymal stem cells and mesoderm-derived tissues. Conditional analysis assessing the effect of the 10 INDELs alongside these SNPs revealed that none of the INDELs reached suggestive significance or showed independent effects.

### PRS performance

In our GWAS, all genome-wide significant variants were located at 4q25, near *PITX2*. To avoid overfitting and utilize well-powered effect estimates, PRSs were built from an independent large-scale Japanese AF GWAS [11] with no sample overlap. Thirty sets of PRS models were created, combining three *P*-value thresholds with 10 LD *r*² thresholds. Among PRS-only models, the highest 5-fold CV-AUCs were consistently achieved using the *P* < 1.0 × 10 ⁻ ⁵ and *r*² > 0.3 threshold set, which included 122 SNPs (S3 Fig). In the test dataset, the LGBM-based PRS-only model achieved the highest AUC (0.702; 95% CI: 0.624–0.770), followed by RF (0.695) and XGB (0.683) (Table 2). All methods showed comparable AUPRC and F1 scores (Table 2). The PRS was standardized using the mean and standard deviation of controls in the training cohort. In the test cohort, the standardized PRS had a mean (± standard deviation) of 0.22 ± 1.01 overall. The mean PRS was significantly higher in cases than in controls (0.50 ± 1.01 vs. −0.16 ± 0.88; *P* = 5.55 × 10 ⁻ ⁶, *t*-test). Calibration performance was assessed using the Brier score in the test dataset. The Brier scores were 0.224 for RF, 0.226 for XGB, and 0.228 for LGBM, indicating similar overall predictive error across models.

**Table 2:** Evaluation of Different Models under the Conditions *P* < 1 × 10 ⁻ ⁵ and *r*² > 0.3 Threshold in the Test Dataset.

Model	AUC (95% CI)	AUPRC	F1
**RF**			
PRS-only model	0.695 (0.619–0.766)	0.763	0.701
Additive model	0.727 (0.656–0.796)	0.774	0.743
Multiplicative model	0.703 (0.630–0.776)	0.761	0.716
**XGB**			
PRS-only model	0.683 (0.606–0.755)	0.749	0.716
Additive model	0.737 (0.668–0.804)	0.781	0.748
Multiplicative model	0.715 (0.643–0.785)	0.769	0.733
**LGBM**			
PRS-only model	0.702 (0.624–0.770)	0.776	0.698
Additive model	0.704 (0.630–0.775)	0.779	0.762
Multiplicative model	0.700 (0.604–0.756)	0.716	0.736

AUC, area under the curve; AUPRC, area under the precision-recall curve; CI, confidence interval; LGBM, light gradient boosting machine; PRS, polygenic risk score; RF, random forest; XGB, extreme gradient boosting. The F1 score was calculated as 2 × (precision × recall)/ (precision + recall). All metrics were computed using scikit-learn v1.6.1 in Python v3.12.9.

**Fig 3:**
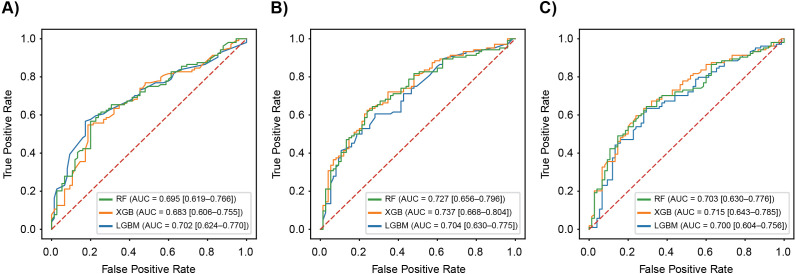
Receiver Operating Characteristic Curves for Three Machine Learning Models in the Test Dataset: (A) PRS-only, (B) Additive, and (C) Multiplicative Models. Curves are shown for RF, XGB, and LGBM; AUCs (95% confidence intervals) are indicated in the legends. AUC, area under the curve; LGBM, light gradient boosting machine; RF, random forest; XGB, extreme gradient boosting.

For additive models incorporating clinical variables, the highest test AUC was achieved using XGB (0.737; 95% CI: 0.668–0.804), followed by RF (0.727) and LGBM (0.704) (Table 2). Minor improvements in AUPRC and F1 scores indicated modest benefits from adding clinical variables. As a sensitivity analysis, clinical-only models achieved AUCs of 0.709 (RF), 0.700 (XGB), and 0.671 (LGBM) in the test dataset (S4 Table). Compared to the clinical-only models, adding PRS to the clinical covariates yielded a modest but consistent improvement in test AUC (Table 2). Multiplicative models, including interaction terms between PRS and clinical variables, showed the best performance using XGB (AUC: 0.715; 95% CI: 0.643–0.785), followed by RF (0.703) and LGBM (0.700) (Table 2). Receiver operating characteristic curves for the test dataset are shown in Fig 3 for the PRS-only, additive, and multiplicative models.

### SHAP-based feature contributions

**Fig 4:**
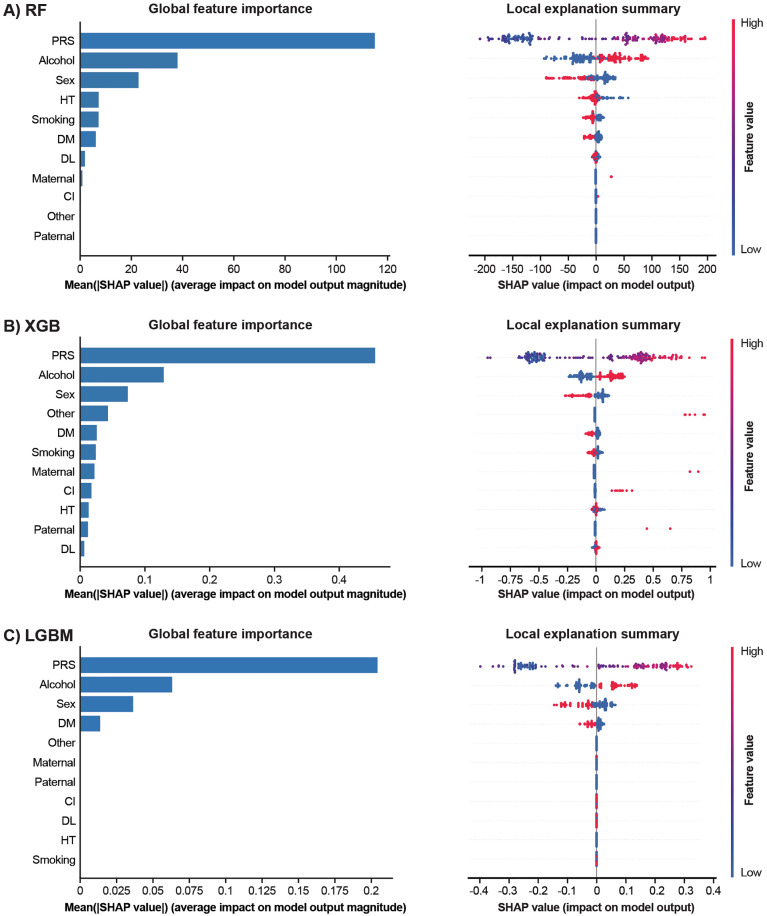
Global and Local SHAP Summary Plots for the Additive Model across Three Machine Learning Algorithms. SHAP global feature importance bar plots (left) and local explanation summary beeswarm plots (right) are shown for **(A)** RF, **(B)** XGB, and **(C)** LGBM. In each model, features are ranked by their mean SHAP values, with the same ranking applied in both bar and beeswarm plots. The bar plots indicate the average contribution of each feature to the magnitude of the model output. In the beeswarm plots, each dot represents one individual in the test dataset. The X-axis shows the SHAP value, indicating the positive or negative contribution of the features to the prediction. Colors represent feature values: red, high; blue, low. Vertical dispersion indicates the density of SHAP values across individuals. CI, cerebral infarction; DL, history of dyslipidemia; DM, history of diabetes mellitus; HT, history of hypertension; LGBM, light gradient boosting machine; Maternal, family history in the mother; Other, family history in other relatives; Paternal, family history in the father; PRS, polygenic risk score; RF, random forest; SHAP, SHapley Additive exPlanations; XGB, extreme gradient boosting.

**Fig 5:**
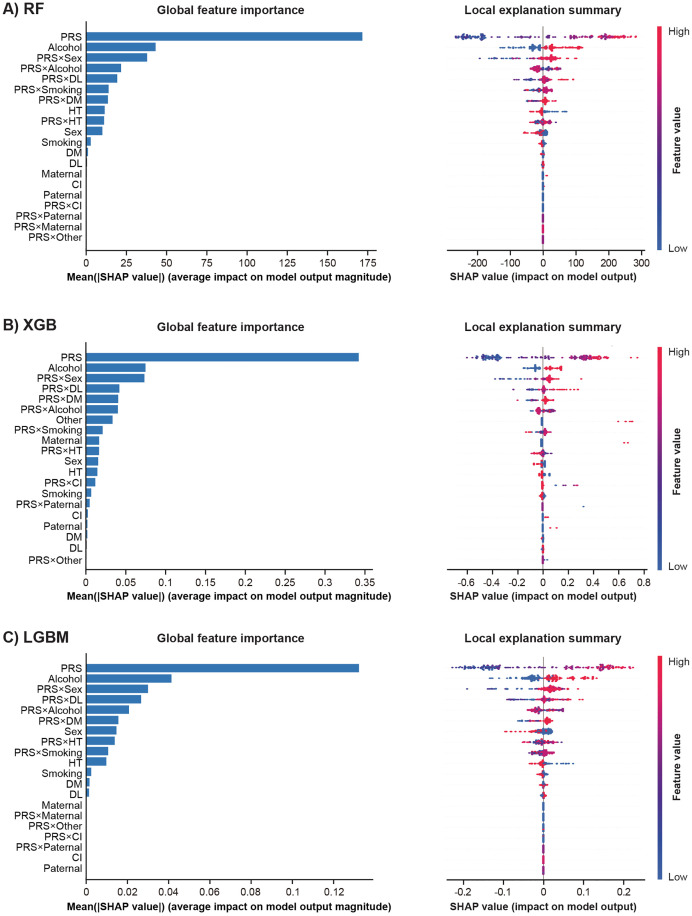
Global and Local SHAP Summary Plots for the Multiplicative Model across Three Machine Learning Algorithms. SHAP global feature importance bar plots (left) and local explanation summary beeswarm plots (right) are shown for **(A)** RF, **(B)** XGB, and **(C)** LGBM. In each model, the top 20 features are ranked by their mean SHAP values, with the same ranking applied in both bar and beeswarm plots. The bar plots indicate the average contribution of each feature to the magnitude of the model output. In the beeswarm plots, each dot represents one individual in the test dataset. The X-axis shows the SHAP value, indicating the positive or negative contribution of the features to the prediction. Colors represent feature values: red, high; blue, low. Vertical dispersion indicates the density of SHAP values across individuals. CI, cerebral infarction; DL, history of dyslipidemia; DM, history of diabetes mellitus; HT, history of hypertension; LGBM, light gradient boosting machine; Maternal, family history in the mother; Other, family history in other relatives; Paternal, family history in the father; PRS, polygenic risk score; RF, random forest; SHAP, SHapley Additive exPlanations; XGB, extreme gradient boosting.

To interpret model predictions, SHAP summary plots were generated for each machine learning method under both additive and multiplicative models. Fig 4 shows the global and local SHAP summary plots for the additive models using RF, XGB, and LGBM. In the global feature importance bar plots on the left, PRS consistently ranked as the most influential predictor across all models. The beeswarm plots (Fig 4, right) show the distribution of SHAP values for each feature, illustrating how variations in feature values influence individual predictions. The wide and symmetrical distribution of SHAP values for PRS indicates its strong and consistent effect on PAF risk prediction. Alcohol consumption and sex were the next most important features, although their contributions were smaller than that of PRS. As shown in the beeswarm plots, individuals who reported alcohol consumption exhibited higher SHAP values than non-drinkers, and men had higher predicted risks than women. Fig 5 shows the same plots for the multiplicative models using RF, XGB, and LGBM. In the global feature importance bar plots, PRS again emerged as the most influential predictor across all models. Several interaction terms, such as PRS × sex and PRS × DL, ranked among the top features; however, their contributions were consistently smaller than that of the main PRS effect. The beeswarm plots on the right display the distribution of SHAP values for both main and interaction features. Although PRS exhibited the widest and most symmetrical SHAP distribution, suggesting a strong and consistent impact on PAF risk prediction, interaction terms showed narrower distributions. For instance, in the XGB model, the mean absolute SHAP value for PRS was 0.34, whereas that for PRS × sex was 0.07, indicating a modest contribution of gene–environment interactions compared with the dominant genetic effect.

## Discussion

This study identified genetic variants associated with PAF in a Japanese population and evaluated the predictive utility of PRSs using multiple machine learning models. In addition to confirming the well-established *PITX2* locus on chromosome 4q25 as a major determinant of AF, our findings offer insight into the genetic architecture and risk modeling of PAF as a distinct clinical subtype. This is among the few studies to focus specifically on PAF and integrate interpretable machine learning approaches to quantify the relative contributions of genetic and clinical factors.

Our GWAS identified 82 genome-wide significant variants, all located upstream of *PITX2* on chromosome 4q25. This region has been repeatedly reported as the most robust genetic risk locus for AF across diverse populations, including Japanese cohorts [8,9,11,16,36–39]. In particular, two independent significant SNPs, rs2200732 and rs13122916, were identified with rs2200732 as the lead SNP in our cohort. A previous well-powered GWAS in a Japanese population identified three independently associated variants near *PITX2* (rs2220427, rs6843082, and rs3853445) linked to AF susceptibility [11]. Other GWASs in the Japanese population have reported additional associated SNPs in this region, including rs4611994, rs1906617, and rs6817105 [16,38,39]. Although rs2200732 exceeded the genome-wide significance threshold (*P* < 5 × 10^−8^) in a previous study, it was not identified as an independent signal [11], suggesting that rs2200732 may represent a proxy variant in linkage disequilibrium with previously reported AF-associated lead variants at the 4q25/*PITX2* locus. Furthermore, the rs2200732 region is enriched with monomethylated histone H3 lysine 4, a marker indicative of enhancer activity [38]. However, enhancer reporter assays do not demonstrate significant differences in promoter activity between risk and protective alleles of rs2200732 [38]. Neither SNP showed deleteriousness or cis-eQTL associations with *PITX2*; however, chromatin interaction analysis revealed that nearby SNPs—rs7434417, rs1906591, and rs6843082—were located in enhancer regions that physically interacted with *PITX2* regulatory domains in mesoderm-derived tissues. In particular, rs7434417 and rs6843082 have previously been reported as AF-associated SNPs [9,11,38], whereas rs1906591 has been associated with ischemic and cardioembolic stroke risk [40]. These findings suggest that chromatin architecture at the 4q25 locus mediates regulatory effects on *PITX2* expression and highlight the need for further functional studies to elucidate the biological relevance of this region in PAF pathogenesis.

We constructed 30 sets of PRS models and evaluated their predictive performance using three tree-based machine learning methods: RF, XGB, and LGBM. Although numerous PRSs have been developed for AF and significant associations between PRS and AF risk have been demonstrated, it has been noted that PRS alone has limited clinical utility [15]. We compared three types of models: PRS-only, additive models (PRS + clinical variables), and multiplicative models (additive model + interaction terms). The best-performing PRS-only models achieved AUCs exceeding 0.70 in the test dataset, outperforming previously reported AF-PRS models, which typically yield AUCs of approximately 0.60 in large datasets, such as the UK Biobank [15]. In a Japanese study, weighted genetic risk scores have achieved AUCs of approximately 0.641 [16]. Furthermore, our additive models showed modest improvements in performance, with AUCs up to 0.737, whereas multiplicative models incorporating gene–environment interactions did not offer additional gains. The consistently top-ranked PRS across all models suggests that, among the predictors included in our models, genetic predisposition contributes substantially to PAF risk prediction, even when major cardiometabolic comorbidities (hypertension, diabetes, and dyslipidemia) and lifestyle factors were considered. These findings highlight the importance of evaluating AF subtypes separately, as risk contributions may vary depending on clinical and pathophysiological profiles. As PAF is often asymptomatic and episodic, with a different natural history and treatment response compared with persistent AF, a better understanding of its genetic determinants may facilitate earlier detection and personalized management.

A key strength of our study is the incorporation of SHAP to enhance model interpretability. SHAP provides individual-level feature attributions, enabling the direct comparison of feature contributions across models. Across all modeling approaches, SHAP consistently identified PRS as the most important predictor, with wide and symmetrical distributions indicating strong and consistent contributions to PAF risk prediction. This pattern was observed in both additive and multiplicative models, suggesting robustness to modeling strategy. Among clinical variables, alcohol consumption and sex emerged as the next most influential features in additive models, although their contributions were smaller than that of PRS. Individuals with a history of alcohol consumption exhibited elevated SHAP values, reflecting an increased predicted risk of PAF. Similarly, sex-related patterns indicated that male participants showed a higher predicted risk than female participants, consistent with prior AF epidemiological findings [41,42]. In contrast, interaction terms in multiplicative models, such as PRS × sex or PRS × DL, did not surpass the main effect of PRS in either SHAP values or model performance. For example, in the XGB multiplicative model, PRS had a mean absolute SHAP value of 0.34, whereas PRS × sex had only 0.07. This quantitative ranking reinforces the conclusion that the explicit modeling of gene–environment interactions offers limited additional value. Previous studies have shown that incorporating covariates, such as age, sex, BMI, and the CHARGE-AF score, can improve model performance, achieving C-indices or AUCs of approximately 0.72–0.83 [15,43,44]. Similar improvements have been observed in Japanese and East Asian cohorts after adjusting for age, sex, genotype array, BMI, hypertension, and major principal components [17,45]. Our results for PAF are consistent with these findings. However, owing to limitations in available data at diagnosis, we were unable to include age of onset, BMI, or CHARGE-AF score, which have been shown to improve prediction accuracy in previous studies. The inclusion of these variables could enhance future models. Moreover, previous studies have demonstrated an association between PRS and age at onset, reporting that individuals with higher PRS values tend to develop AF at younger ages [16,18,46,47]. In the present study, age at diagnosis was known for some participants, with most diagnosed after the age of 60 years. Further studies, including younger patients with PAF, are needed to evaluate age-specific predictive accuracy and clarify the role of PRS across age strata.

This study has several strengths, including the use of a well-characterized Japanese cohort, application of multiple tree-based machine learning algorithms, and incorporation of interaction modeling and SHAP-based interpretability analyses. However, certain limitations should be acknowledged. First, external validation in an independent Japanese cohort was not performed. Additionally, owing to the modest sample size, this study may have been underpowered to detect additional genome-wide significant loci beyond the 4q25/*PITX2* region, particularly for variants with small effect sizes or low allele frequencies. To confirm the reliability and reproducibility of our findings, larger studies and validation in independent cohorts will be required. Second, as the control group comprised individuals recruited from cardiology departments, it does not fully represent the general population, potentially introducing selection bias. Consequently, the generalizability of our findings may be limited. In addition, because PAF can be intermittent and asymptomatic, undiagnosed PAF among controls cannot be completely excluded. Future studies, incorporating population-based controls or external cohorts, would aid in addressing this limitation and enable sensitivity analyses to assess the effect of control selection on risk estimation. Third, the PRS was derived from AF-associated loci identified in previous studies, which may not fully capture the genetic risk specific to PAF. Future analyses using PAF-specific GWAS summary statistics, once available, may improve predictive accuracy. In addition, because variants with MAF < 0.01 were excluded during quality control in this GWAS with imputation, we did not evaluate rare-variant associations; further studies will be needed to assess the contribution of rare variants to PAF. Fourth, the use of a single train/test data split restricts a comprehensive assessment of variability in model performance. Additionally, because the test dataset comprised only 10% of the cohort, performance estimates may be unstable. Although five-fold cross-validation was applied within the training dataset for hyperparameter tuning, a fixed train/test data split was maintained to prevent information leakage and ensure fair comparisons across PRS thresholds and machine learning models. While this approach favors comparability, it may underrepresent performance variability. Even though we reported bootstrap-based confidence intervals, performance may still be sensitive to the specific random split. Future studies should assess model stability using repeated resampling or nested cross-validation in larger cohorts and, importantly, validate the models in independent external cohorts. Finally, as this study focused exclusively on a Japanese population, the external generalizability of our findings to other ethnic groups remains untested. Further studies are needed to validate these results in diverse populations to establish their broader applicability.

From a clinical perspective, our findings suggest that PRS could serve as a valuable component in the early risk stratification of PAF, particularly among individuals who do not exhibit conventional clinical risk factors. Given the intermittent and frequently asymptomatic nature of PAF, genetics-based risk models may facilitate proactive screening strategies and enable earlier detection and intervention.

## Conclusions

The present study demonstrated that variants near the *PITX2* locus strongly influenced PAF risk in the Japanese population and that PRSs derived from AF-associated loci offered robust predictive value even when used independently. Incorporating clinical variables in additive models yielded modest improvements, whereas multiplicative models offered minimal incremental benefit. SHAP-based interpretability analyses consistently identified PRS as the most influential predictor among the clinical variables included in our models. However, some established clinical predictors, particularly BMI at the time of PAF diagnosis and validated clinical risk scores, were not available for all participants. Incorporating these parameters into future models and validating them with external cohorts could enhance individual-level risk prediction and increase clinical relevance.

## Supporting information

S1 Fig Flowchart of Participant and Variant Selection during Quality Control and Genotype Imputation. In total, 2,604 participants were genotyped. Of these, 764 participants based on clinical criteria and 58 participants based on genotype-based quality control were excluded, retaining 1,782 participants (cases: 1,038; controls: 744) for the final analysis. In total, 443,911 SNPs were identified in these participants after removing 215,273 variants based on quality control criteria. Following subsequent genotype imputation using the 1000 Genomes Phase 3 reference panel [25], variants with low imputation quality (Rsq < 0.3), low minor allele frequency (<0.01), or duplication were excluded. Finally, 8,094,202 SNPs were retained for GWAS and PRS analyses. AF, atrial fibrillation; CAD, coronary artery disease; HF, heart failure; PAF, paroxysmal atrial fibrillation; PCA, principal component analysis; PRS, polygenic risk score. a: 5 cases and 2 controls with 2 or 3 overlapping exclusions.
(PDF)

S2 Fig Regional Plot of Chromosome 4 with Surrounding Independent Significant SNPs. Independent significant SNPs are represented using purple diamonds. Colors indicate linkage disequilibrium (*r*^2^) with independent significant SNPs. (A) rs2200732. (B) rs13122916. SNP, single-nucleotide polymorphism.
(PDF)

S3 Fig Cross-validated AUC Heatmaps for PRS-only Models using Three Machine Learning Methods. Heatmaps showing the cross-validated AUC values for PRS-only models across combinations of *P*-value and linkage disequilibrium *r*^2^ thresholds. Three machine learning models are shown: RF, XGB, and LGBM. Each heatmap displays AUC values derived from five-fold cross-validation in the training dataset. Rows correspond to linkage disequilibrium *r*^2^ thresholds (no clumping and 0.9 to 0.1) and columns represent *P*-value thresholds (5 × 10^−8^, 1 × 10^−5^, and 1 × 10^−4^). Darker shades indicate higher AUCs. The numeric values within each cell represent the mean AUC obtained for that specific parameter combination. AUC, area under the curve; LGBM, light gradient boosting machine; PRS, polygenic risk score; RF, random forest; XGB, extreme gradient boosting.
(PDF)

S1 Table Hyperparameter Search Spaces and Fixed Settings. Hyperparameter tuning was performed on the training dataset only using Bayesian optimization with Optuna (Tree-structured Parzen Estimator; five-fold stratified cross-validation; 100 trials per algorithm; seed = 42), maximizing cross-validated area under the curve. LGBM, light gradient boosting machine; RF, random forest; XGB, extreme gradient boosting.
(XLSX)

S2 Table Variants associated with Paroxysmal Atrial Fibrillation reaching Genome-wide Significance. A1, risk allele; A2, reference allele; BETA, regression coefficient; Chr, Chromosome; L95, lower 95% confidence interval; U95, upper 95% confidence interval. Chromosome position (GRCh37/hg19).
(XLSX)

S3 Table Variants associated with Paroxysmal Atrial Fibrillation reaching Suggestive Significance. A1, risk allele; A2, reference allele; BETA, regression coefficient; Chr, Chromosome; L95, lower 95% confidence interval; U95, upper 95% confidence interval. Chromosome position (GRCh37/hg19).
(XLSX)

S4 Table Performance of clinical-covariate–only models (excluding PRS) in the test dataset. AUC, area under the curve; AUPRC, area under the precision-recall curve; CI, confidence interval; LGBM, light gradient boosting machine; RF, random forest; XGB, extreme gradient boosting. The F1 score was calculated as 2 × (precision × recall)/(precision + recall). All metrics were computed using scikit-learn v1.6.1 in Python v3.12.9.
(XLSX)
