# Supplementary material for: Genetic variants and polygenic risk scores associated with paroxysmal atrial fibrillation in the Japanese population
Source: PLoS One. 2026 May 4;21(5):e0344360. doi: 10.1371/journal.pone.0344360 (PMC13138623; doi:10.1371/journal.pone.0344360)
Supplement: S1 Fig — In total, 2,604 participants were genotyped. Of these, 764 participants based on clinical criteria and 58 participants based on genotype-based quality control were excluded, retaining 1,782 participants (cases: 1,038; controls: 744) for the final analysis. In total, 443,911 SNPs were identified in these participants after removing 215,273 variants based on quality control criteria. Following subsequent genotype imputation using the 1000 Genomes Phase 3 reference panel [25], variants with low imputation quality (Rsq < 0.3), low minor allele frequency (<0.01), or duplication were excluded. Finally, 8,094,202 SNPs were retained for GWAS and PRS analyses. AF, atrial fibrillation; CAD, coronary artery disease; HF, heart failure; PAF, paroxysmal atrial fibrillation; PCA, principal component analysis; PRS, polygenic risk score. a: 5 cases and 2 controls with 2 or 3 overlapping exclusions. (PDF) [file pone.0344360.s001.pdf]

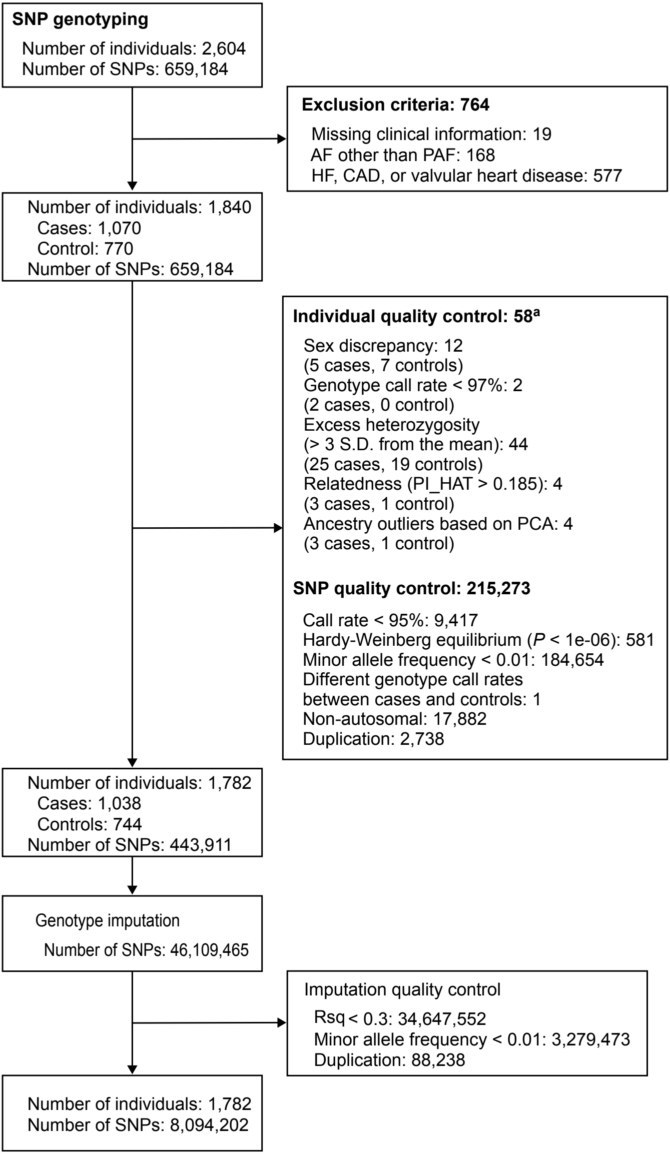

SNP genotyping
Number of individuals: 2,604
Number of SNPs: 659,184
Exclusion criteria: 764
Missing clinical information: 19
AF other than PAF: 168
HF, CAD, or valvular heart disease: 577
Number of individuals: 1,840
Cases: 1,070
Control: 770
Number of SNPs: 659,184
Individual quality control: 58[a]
Sex discrepancy: 12
(5 cases, 7 controls)
Genotype call rate < 97%: 2
(2 cases, 0 control)
Excess heterozygosity
(> 3 S.D. from the mean): 44
(25 cases, 19 controls)
Relatedness (PI_HAT > 0.185): 4
(3 cases, 1 control)
Ancestry outliers based on PCA: 4
(3 cases, 1 control)
SNP quality control: 215,273
Call rate < 95%: 9,417
Hardy-Weinberg equilibrium (P < 1e-06): 581
Minor allele frequency < 0.01: 184,654
Different genotype call rates
between cases and controls: 1
Non-autosomal: 17,882
Duplication: 2,738
Number of individuals: 1,782
Cases: 1,038
Controls: 744
Number of SNPs: 443,911
Genotype imputation
Number of SNPs: 46,109,465
Imputation quality control
Rsq < 0.3: 34,647,552
Minor allele frequency < 0.01: 3,279,473
Duplication: 88,238
Number of individuals: 1,782
Number of SNPs: 8,094,202
