# Supplementary figures and images for: Genetic variants and polygenic risk scores associated with paroxysmal atrial fibrillation in the Japanese population
Source: PLoS One. 2026 May 4;21(5):e0344360. doi: 10.1371/journal.pone.0344360 (PMC13138623; doi:10.1371/journal.pone.0344360)

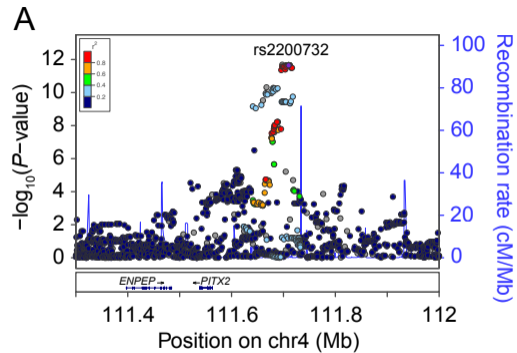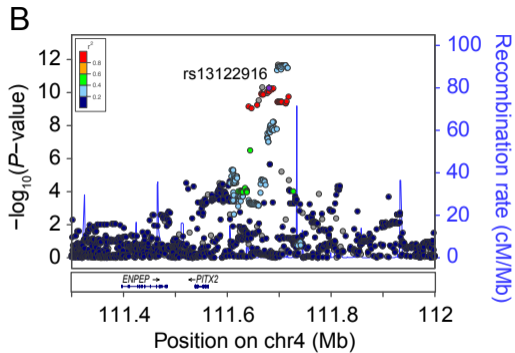

Supplement: S2 Fig — Independent significant SNPs are represented using purple diamonds. Colors indicate linkage disequilibrium (r2) with independent significant SNPs. (A) rs2200732. (B) rs13122916. SNP, single-nucleotide polymorphism. (PDF) [file pone.0344360.s002.pdf]

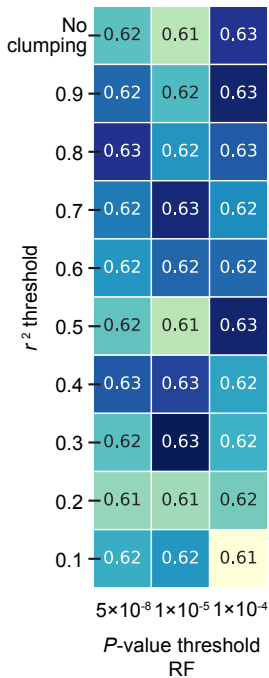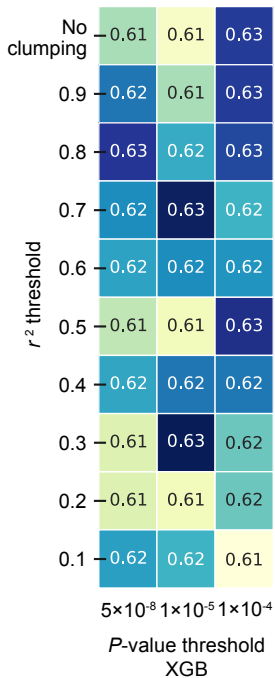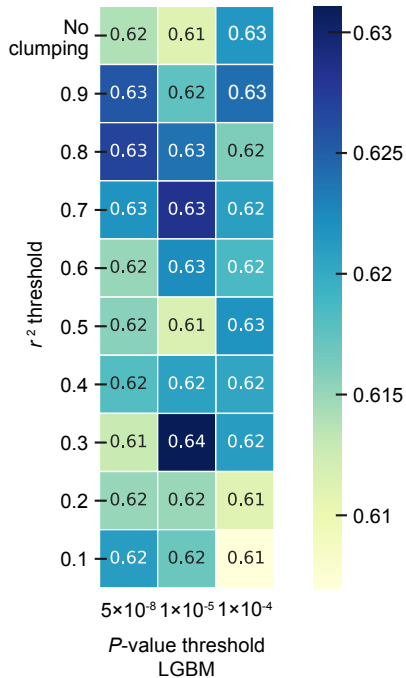

Supplement: S3 Fig — Heatmaps showing the cross-validated AUC values for PRS-only models across combinations of P-value and linkage disequilibrium r2 thresholds. Three machine learning models are shown: RF, XGB, and LGBM. Each heatmap displays AUC values derived from five-fold cross-validation in the training dataset. Rows correspond to linkage disequilibrium r2 thresholds (no clumping and 0.9 to 0.1) and columns represent P-value thresholds (5 × 10^−8^, 1 × 10^−5^, and 1 × 10^−4^). Darker shades indicate higher AUCs. The numeric values within each cell represent the mean AUC obtained for that specific parameter combination. AUC, area under the curve; LGBM, light gradient boosting machine; PRS, polygenic risk score; RF, random forest; XGB, extreme gradient boosting. (PDF) [file pone.0344360.s003.pdf]
